# Appropriate Management of Thrombotic Risk in Patients With Primary Immune Thrombocytopenia in the UK: A Modified Delphi Consensus

**DOI:** 10.1002/jha2.70134

**Published:** 2025-09-03

**Authors:** Charlotte Bradbury, Jecko Thachil, Matthew McWilliams, Will A. Lester

**Affiliations:** ^1^ Department of Haematology University of Bristol Bristol UK; ^2^ Manchester Royal Infirmary Manchester UK; ^3^ SOBI Ltd Cambridge UK; ^4^ University Hospitals Birmingham Birmingham UK

**Keywords:** Delphi consensus, primary immune thrombocytopenia, thrombosis, UK

## Abstract

**Introduction:**

Immune thrombocytopenia (ITP) is characterised by a low platelet count and increased risk of bleeding. Recent research has also proposed that having ITP increases thrombosis risk. Moreover, certain ITP treatments have been associated with an increased risk of thrombosis. This Delphi study aims to assess haematologist opinion regarding aspects of optimise thrombotic risk management in primary ITP in the UK.

**Methods:**

The methodology employed a modified Delphi process with two rounds of evaluation from a panel of experts. A literature review on the topic of primary ITP generated input to a steering group of three experts from the UK attended a virtual meeting in May 2024. During this meeting, and guided by an independent facilitator, the group identified five main domains. From these, 42 statements were agreed and developed into an online survey for testing with a wider panel of peers.

**Results:**

Overall, 33 statements achieved consensus agreement, and one statement did not achieve consensus. Eight scenario statements were included to identify preferable treatment options among healthcare professionals.

**Conclusion:**

Based on the agreement levels achieved, the steering group formulated a set of recommendations to optimise the management of thrombotic risk in patients with primary ITP in the UK.

**Trial Registration:**

The authors have confirmed clinical trial registration is not needed for this submission.

## 1 Introduction

Immune thrombocytopenia (ITP) is a rare condition characterised by low platelet count (typically less than 100 × 10^9^/L) and increased haemorrhagic events [1]. The incidence of ITP is approximately 3 cases per 100,000 person‐years worldwide [2].

ITP is classified as either primary or secondary. Primary ITP (pITP) is idiopathic, lacking an identifiable underlying cause, whereas secondary ITP is associated with another cause or condition [1]. In the UK, data from 2003 and 2014 indicates an increasing incidence by approximately 4.3% per year, particularly among young women and elderly men [3]. As more than 50% of patients are at risk of developing chronic ITP, long‐term management is often required, which places a significant burden on the healthcare system [4].

The clinical presentation of ITP includes various symptoms such as skin purpura, nosebleeds, gastrointestinal, urogenital and vaginal bleeding. Severe bleeding events, such as intracranial haemorrhages, are linked to an increased risk of mortality. Low platelet levels are also associated with fatigue, which has a substantial impact on the quality of life for most patients [5].

The primary goal of treatment in pITP is to maintain platelet counts at a safe level to prevent haemorrhagic events [6]. Treatment for thrombocytopenia is recommended when the platelet count falls below 20 × 10^9^/L, although the threshold for initiating treatment may vary according to individual bleeding risk [7].

The treatment landscape has changed considerably in the last decade, with more evidence‐based treatment options now available, such as thrombopoietin receptor agonists (TPO‐RAs) and spleen tyrosine kinase inhibitors [6, 8]. Agents targeting other molecular pathways are also currently in development [9].

Patients with ITP are also at increased risk of developing thrombosis [10]. While this seems counterintuitive given the role of platelets in the formation of blood clots, several factors may underlie the risk of thrombosis in ITP patients [10]. Moreover, certain treatments, particularly TPO‐RAs, have been associated with an expected increased risk of thrombosis, which raises questions about how best to manage thrombotic risk in patients with ITP [11, 12]. This is compounded by a lack of guidance on appropriate management of thromboembolic events (TEEs) in ITP within current available guidelines. The aim of this modified Delphi study was to gather expert opinions on optimising the management of thrombotic risk in patients with pITP in the UK.

## 2 Materials and Methods

**FIGURE 1:**
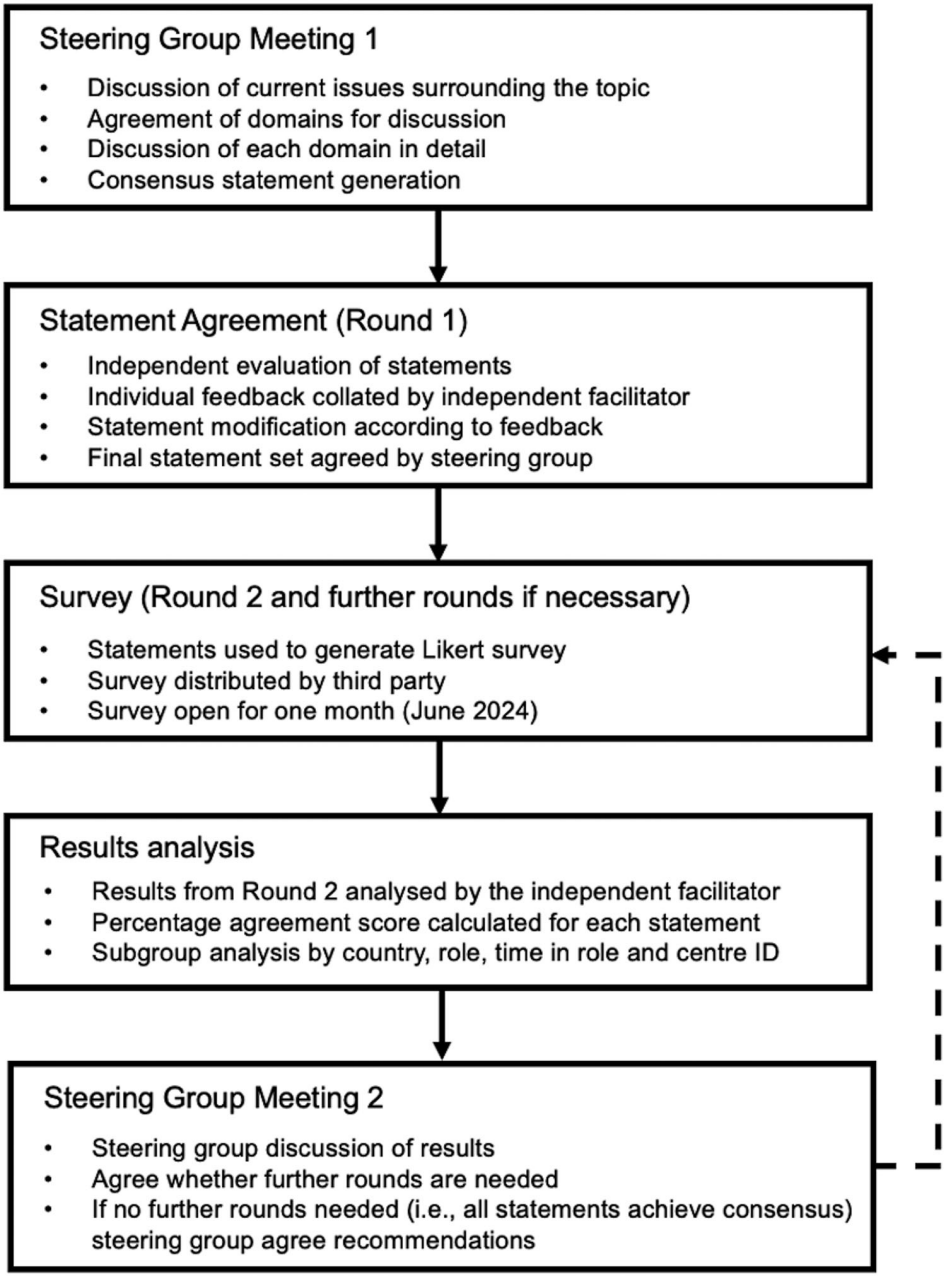
Modified Delphi study design.

The present study employed a modified Delphi methodology to arrive at several consensus statements on the appropriate management of thrombosis in ITP (Figure 1). The modified Delphi process uses a systematic approach design to assist a group of experts reach consensus through a blend of in‐depth discussions and anonymous, iterative surveys with a wider panel. This method allows for the validation of opinions of a small steering group with a much broader, relevant panel of respondents. Unlike other Delphi methods that rely solely on questionnaires, this modified approach incorporated steering group meetings to allow real‐time discussion, exchange of opinions and statement development [13].

An initial literature review was conducted on PubMed, Cochrane and Google Scholar. Search terms included but were not limited to: ‘immune thrombocytopenia,’ ‘chronic ITP,’ ‘TPO‐RA,’ ‘Patient burden’ and ‘Guidelines.’ The findings from this review were used to inform an expert steering group of three UK‐based haematology experts to discuss the current barriers and opportunities in the management of thrombotic risk in patients with pITP. The steering group members were selected based on their professional roles, experience and publication history. All members of the steering group attended both meetings in the modified Delphi process but did not participate in the wider panel. During this meeting, the group identified five main domains of focus:

- Management of patients already on antithrombotic treatment who present with ITP
- Risk assessment of newly diagnosed patients with ITP
- Managing new acute thrombotic events in patients with ITP
- Optimising a multi‐disciplinary (MDT) approach to care
- Scenario testing of platelet thresholds in treatment decisions

Based on these domains, consensus statements were proposed by the group during the initial meeting. The statements were then independently reviewed by the individual group members as either ‘accept,’ ‘remove,’ or ‘reword with suggested changes.’ Recommendations were accepted based on a simple majority. This constituted the initial round of consensus. In addition, the group defined eight ‘scenario statements’ designed to provide understanding of how a four‐point Likert survey was developed (‘*strongly agree*,’ ‘*agree*,’ ‘*disagree*’ and ‘*strongly disagree*’) to test the statements, which was distributed to a wider Delphi panel for testing by a third party, M3 Global. M3 Global were solely responsible for survey administration and raw data collection, they did not have influence over study design, data analysis or results interpretation. M3 Global holds a large panel of healthcare professionals, each respondent received a nominal and universal compensation for their participation. Respondents were recruited according to the following criteria:

- Current role of haematologist and/or pITP specialist
- Respondents not based at one of the 31 ITP specialist centres must be currently in role of haematologist
- Either based in one of 31 ITP centres or other hospitals (see above point)

A statement of consent was included at the start of the survey. Following standard practice for Delphi studies, the anonymity of responders was planned into the study design [14]. Three demographic screening questions were used at the start of the survey, once qualified for the survey, the participants were directed to give their responses to the 42 statements. No personal information beyond demographic data (current role, time in the current role, country and centre ID) was captured. Anonymity and confidentially measures are important in qualitative and quantitative studies to protect participant privacy and to provide a platform for panellists to provide feedback openly and without any concern over repercussion [15].

Stopping criteria were agreed as a target of 50 responses, and at least 90% of statements achieving consensus [16], with consensus defined as 75% agreement for each statement. These thresholds have been used in previous consensus studies [17]. Using Microsoft Excel, completed surveys were analysed to produce an arithmetic agreement score for each statement. The responses were aggregated to provide a mean agreement score (MAS) (i.e., the number of responses expressing agreement as a percentage of the overall number of responses for each statement).

If the stopping criteria were met, further iterations of survey were deemed unnecessary. Completed surveys were analysed by the independent facilitator to produce an arithmetic agreement score for each statement. This was then reviewed by the members of the steering group to agree next steps. Controlled qualitative feedback from the steering group over email when refining the initial statements and provided during virtual discussion when presented with the results. If stopping criteria were not met, a summary of previous round results would be provided to panel members during subsequent rounds.

### 2.1 Patient and Public Involvement

There was no patient or public involvement. This study provides a basis to inform clinical guidelines for ITP management and, while a recognised limitation, the decision was made to not include patient and public involvement (PPI) at this stage in the process owing to the concentration on clinician‐focused guidelines. There is inherent possibility to introduce PPI to shape an integrated pathway which builds on the recommendations presented herein.

## 3 Results

Note, individuals statements are referred to below by their domain and statement number (e.g., Statement 1 = A1, Statement 9 = B9, etc.)

At the end of Round 1 a final set of 42 statements was agreed. This included 34 single sentence statements plus eight scenario‐based questions. Completed surveys were received from 46 haematologists, and all were included in the final analysis.

A total of 36 respondents reported above 6 years of experience in role, seven reported 4–5 years, and three had 1–3 years (Figure S1). Most respondents were based in England (*n* = 41), with two based in Wales and Scotland each, and one respondent from Northern Ireland (Figure S2). Distribution of respondents by centre ID is represented in Figure S3.

**FIGURE 2:**
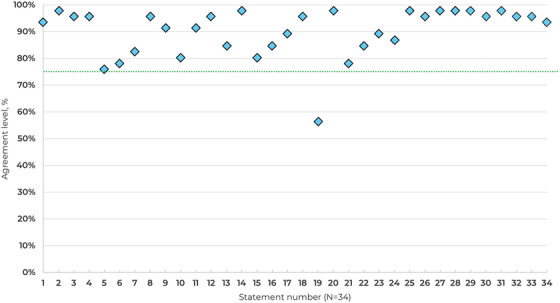
Consensus agreement levels by statement. The threshold for consensus is depicted by the green line (75%).

At the end of Round 2 consensus agreement was achieved in 33/34 statements, of which 21 achieved ≥ 90% agreement (Table 1, Figure 2). Distribution of consensus scores by agreement level is shown in Figure S4. The eight scenario statements were analysed separately to identify preferable treatment options among haematologists (Table 2, Figures S5 and S6).

**TABLE 1:** Defined consensus statements and corresponding levels of agreement (all numbers rounded to the nearest whole number).

No	Statement	Strongly agree	Tend To agree	Tend To disagree	Strongly disagree	Agreement
**Domain A: Management of patients already on antithrombotic treatment who present with ITP**						
1	Assessment of thrombotic risk should be done in conjunction with the relevant specialist to determine the risks of reducing/pausing antithrombotic treatment	65%	28%	4%	2%	**93%**
2	Assessing bleeding risk is vital to determine the risk of continuing anticoagulation	85%	13%	0%	2%	**98%**
3	Assessment of bleeding risk should include platelet count, clinical phenotype, type and dose of antithrombotic, and the presence of active bleeding	74%	22%	2%	2%	**96%**
4	When platelet count is low (< 50 × 10^9^/L), a decision needs to be made whether to pause, reduce or modify antithrombotic treatment	72%	24%	2%	2%	**96%**
5	Uncertainty exists about which patients to pause antithrombotic medication in patients with ITP	41%	35%	24%	0%	**76%**
6	Uncertainty exists about when to restart antithrombotic medication in patients with ITP	33%	46%	22%	0%	**78%**
7	Uncertainty exists about when to modify antithrombotic medication in patients with ITP	37%	46%	15%	2%	**83%**
8	A plan for restarting antithrombotic treatment once the platelet count has improved should be in place for all patients	74%	22%	4%	0%	**96%**
**Domain B: Risk assessing newly diagnosed patients with ITP (newly diagnosed – thrombotic risk assessment)**						
9	Assessment of thrombotic risk in ITP should be done in conjunction with a relevant haematology ITP specialist	39%	52%	2%	7%	**91%**
10	Arterial and venous thrombotic risk factors should be assessed in newly diagnosed ITP patients	46%	35%	17%	2%	**80%**
11	Thrombotic risk is one factor to consider when choosing an ITP directed treatment, but in general, thrombotic risk factors should not contraindicate the use of a TPO‐RA	43%	48%	7%	2%	**91%**
12	Arterial and venous thrombotic risk factors should be regularly reassessed (e.g., at times of increased risk such as surgery) as thromboprophylaxis may be indicated even in the presence of thrombocytopaenia	50%	46%	4%	0%	**96%**
13	Alternative immunomodulatory treatments for ITP (e.g. rituximab) may be preferred in patients with strongly positive antiphospholipid (APS) serology	43%	41%	13%	2%	**85%**
14	Modifiable risk factors should be reviewed, and the benefits of changing them should be weighed against the risk of doing this	50%	48%	2%	0%	**98%**
15	The risk and downsides of stopping HRT or oral contraception may outweigh the thrombotic risk of continuing	24%	57%	20%	0%	**80%**
16	Antiphospholipid syndrome screening, including anticardiolipin antibodies (aCL); lupus anticoagulant (LA); anti‐beta2‐glycoprotein‐1 (anti‐B2GP1), should be carried out in selected newly diagnosed patients as it may influence choice of subsequent therapy for ITP	41%	43%	13%	2%	**85%**
17	ITP treatments carry some level of thrombotic risk, but this is only one factor to consider when deciding the individual treatment approach	48%	41%	9%	2%	**89%**
18	Thrombocytopenia does not protect patients from thrombosis	43%	52%	4%	0%	**96%**
19	There is no evidence that high platelet counts on TPO‐RA treatment correlate with thrombotic risk	11%	46%	30%	13%	**57%**
**Domain C: Managing new acute thrombotic events in patients with ITP**						
20	Assessment of bleeding risk versus the severity of thrombotic event is vital	63%	35%	2%	0%	**98%**
21	Withdrawing/pausing effective ITP therapy (e.g. TPO‐RA) should be avoided due to the risk of rebound thrombocytopenia	30%	48%	17%	4%	**78%**
22	Withdrawing/pausing effective ITP therapy (e.g. TPO‐RA) should be avoided as managing an acute thrombotic event relies on a haemostatic platelet count	30%	54%	13%	2%	**85%**
23	In general, patients can be treated as per standard care/indication if the platelet count is ≥ 50 × 10^9^/L and the patient is not bleeding	59%	30%	9%	2%	**89%**
24	A platelet count < 50 × 10^9^/L is not necessarily a contraindication for antithrombotic treatment	35%	52%	11%	2%	**87%**
25	Assessing bleeding risk is important to decide the risk of antithrombotic treatment. Platelet count is one factor to consider but other bleeding risks should also be assessed including previous bleeding, current bleeding, renal failure, type and dose of antithrombotic treatment indicated	67%	30%	2%	0%	**98%**
26	While the platelet count is < 50 × 10^9^/L, a decision needs to be made about whether to initiate full antithrombotic treatment, withhold or modify the dose of antithrombotic treatment	63%	33%	4%	0%	**96%**
27	A decision needs to be made as to whether ITP‐directed therapy is needed to increase the platelet count	72%	26%	2%	0%	**98%**
28	A plan for restarting antithrombotic treatment once the platelet count has improved should be in place for all patients	67%	30%	2%	0%	**98%**
**Domain D: Optimising a multi‐disciplinary (MDT) approach to care**						
29	Education on thrombotic management in ITP should be available for the care team	70%	28%	2%	0%	**98%**
30	Support for clinicians regarding decision making in challenging cases needs to be accessible via expert centres	70%	26%	4%	0%	**96%**
31	Support for patients, including via nursing and through access to psychology is desirable	59%	39%	2%	0%	**98%**
32	Nurses play an important/critical role in the MDT	59%	37%	4%	0%	**96%**
33	The role of the MDT is important when making treatment decisions	59%	37%	4%	0%	**96%**
34	Involving patients in the treatment decision is important	65%	28%	7%	0%	**93%**

**TABLE 2:** Scenario testing statements and corresponding levels of agreement (all numbers rounded to the nearest whole number).

Domain E: Scenario testing of platelet thresholds in treatment decisions (Matrix of factors and platelet thresholds to establish consensus regarding when to treat)						
No.	Clinical scenario 1	Full dose anticoagulant	Half dose anticoagulant	Prophylactic anticoagulant	No anticoagulant	Other
35	Patient with ITP and platelet count of 15 × 10^9^/L develops new DVT. Not actively bleeding	0%	26%	22%	39%	13%
36	Patient with ITP and platelet count of 25 × 10^9^/L develops new DVT. Not actively bleeding	4%	43%	35%	13%	4%
37	Patient with ITP and platelet count of 35 × 10^9^/L develops new DVT. Not actively bleeding	24%	54%	17%	2%	2%
38	Patient with ITP and platelet count of 45 × 10^9^/L develops new DVT. Not actively bleeding	50%	41%	4%	2%	2%

No.	Clinical scenario 2	Continue DAPT	Continue aspirin monotherapy	Continue clopidogrel monotherapy	Stop DAPT until platelets improve
39	A 55‐year‐old patient with ITP receives IVIg for a viral triggered exacerbation. They have an MI while platelet count is normal. They have LAD PCI and are started on clopidogrel and aspirin. Three months later, they had a further exacerbation of ITP, and platelet count is 15 × 10^9^/L. There is no bleeding. You give treatment to boost the platelet count. Antiplatelet treatment options (assuming close monitoring)	4%	35%	7%	54%
40	A 55‐year‐old patient with ITP receives IVIg for a viral triggered exacerbation. They have an MI while platelet count is normal. They have LAD PCI and are started on clopidogrel and aspirin. Three months later, they had a further exacerbation of ITP, and platelet count is 25 × 10^9^/L. There is no bleeding. You give treatment to boost the platelet count. Antiplatelet treatment options (assuming close monitoring)	9%	57%	11%	24%
41	A 55‐year‐old patient with ITP receives IVIg for a viral triggered exacerbation. They have an MI while platelet count is normal. They have LAD PCI and are started on clopidogrel and aspirin. Three months later, they had a further exacerbation of ITP, and platelet count is 35 × 10^9^/L. There is no bleeding. You give treatment to boost the platelet count. Antiplatelet treatment options (assuming close monitoring)	35%	43%	15%	7%
42	A 55‐year‐old patient with ITP receives IVIg for a viral triggered exacerbation. They have an MI while platelet count is normal. They have LAD PCI and are started on clopidogrel and aspirin. Three months later, they had a further exacerbation of ITP, and platelet count is 45 × 10^9^/L. There is no bleeding. You give treatment to boost the platelet count. Antiplatelet treatment options (assuming close monitoring)	57%	24%	17%	2%

The results demonstrate strong consensus on several aspects of managing thrombotic risk in patients with ITP. A significant majority (A1, 93%) agreed that assessment of thrombotic risk should be agreed through collaboration of specialists to evaluate the risks of adjusting or pausing antithrombotic treatment. Respondents emphasised the importance of assessing bleeding risk to guide decisions about continuing anticoagulation (A2, 98%), and that this assessment should include factors such as platelet count, clinical phenotype, antithrombotic type and dose and any active bleeding (A3, 96%) [18, 19]. In addition, when platelet counts drop below 50 × 10^9^/L, decisions about pausing or modifying antithrombotic treatment are critical (A4, 96%).

Uncertainty remains regarding when to pause (A5, 76%), restart (A6, 78%) or modify (A7, 83%) antithrombotic treatment. However, respondents did agree that a plan for restarting treatment once platelet levels improve should be established for all patients (A8, 96%).

Thrombotic risk assessment should be performed in collaboration with relevant haematology ITP specialists (B9, 91%). Respondents agreed that arterial and venous thrombotic risk factors should be evaluated in newly diagnosed ITP patients (B10, 80%) [12].

While thrombotic risk is an important consideration in choosing ITP‐directed treatments, it should not contraindicate the use of TPO‐RA (B11, 91%). Regular reassessment of thrombotic risk factors is crucial, especially during periods of increased risk of thrombosis, such as surgery, since thromboprophylaxis may be required even in the presence of thrombocytopenia (B12, 96%).

Alternative immunomodulatory treatments, such as rituximab, may be preferred for patients with strongly positive antiphospholipid syndrome (APS) serology (B13, 85%) [20]. A thorough review of modifiable risk factors should be conducted, weighing the benefits of making changes against the risks (B14, 98%).

The risks and downsides of stopping hormone replacement therapy (HRT) or oral contraception may outweigh the thrombotic risks of continuation (B15, 80%). Screening for APS, including tests for anticardiolipin antibodies, lupus anticoagulant and anti‐beta2‐glycoprotein‐1, should be conducted in newly diagnosed patients with ITP. This may help to assess thrombotic risk and influence subsequent therapy choices (B16, 85%) [7, 21].

ITP treatments may carry some level of increase in thrombotic risk, but this is only one factor to consider in determining the appropriate treatment strategy, (B17, 89%). Furthermore, thrombocytopenia does not protect patients from thrombosis (B18, 96%) [22].

Data from TPO‐RA studies indicate that these agents may be associated with a small additional risk of arterial and venous thrombosis [7]. B19 proposed that there was no evidence that high platelet counts on TPO‐RA treatment correlated with thrombotic risk but received only 57% agreement.

Strong agreement was achieved regarding key considerations for managing bleeding and thrombotic risk in patients with ITP. It was agreed that assessing bleeding risk against the severity of a thrombotic event is essential (C20, 98%). Withdrawing or pausing effective ITP therapy, such as TPO‐RAs, should be avoided due to the risk of rebound thrombocytopenia (C21, 78%), and the need to maintain a haemostatic platelet count during acute thrombotic events to enable appropriate antithrombotic treatment (C22, 85%). Participants agreed that patients can generally be treated according to standard care if platelet counts are ≥ 50 × 10^9^/L and there is no active bleeding (C23, 89%), and that a platelet count < 50 × 10^9^/L is not necessarily a contraindication for antithrombotic treatment (C24, 87%).

Bleeding risk assessment should consider multiple factors, including platelet count, bleeding history, renal function and the type and dose of antithrombotic treatment (C25, 98%). Furthermore, when platelet counts are < 50 × 10^9^/L, a decision is needed regarding whether to initiate, withhold or modify antithrombotic therapy (C26, 96%). In addition, a decision is needed on whether ITP‐directed therapy should be used to increase platelet counts (C27, 98%), with a plan in place for restarting antithrombotic treatment once platelet counts improve (C28, 98%).

Respondents (D29, 98%) agreed that education on thrombotic management in important for the care team, and clinicians should have access to expert centres for support in making decisions in challenging cases (96%, S30). Similarly, providing patient support, including nursing and psychological access, is desirable (D31, 98%). The critical role nurses can play in the MDT was recognised along with the importance of the MDT in making treatment decisions (D32, S33, 96% each). Furthermore, patients should be actively involved in their treatment decisions (D34, 93%) [7, 23, 24].

In the first scenario (E35–E38; Figure S5):

- For a patient with a platelet count of 15 × 10⁹/L, the management options for DVT included: no anticoagulant 39%), half‐dose anticoagulant (26%) and prophylactic anticoagulant (22%). Notably, none recommended full‐dose anticoagulants.
- For a patient with a platelet count of 25 × 10⁹/L, the preferences shifted, with 35% favouring prophylactic anticoagulants and 43% selecting half‐dose anticoagulants, while only 4% opted for full‐dose anticoagulants.
- For a platelet count of 35 × 10⁹/L, 54% of responses indicated a preference for half‐dose anticoagulants, while 24% supported full‐dose anticoagulants.
- For a platelet count of 45 × 10⁹/L, there was a notable shift toward full‐dose anticoagulants, with 50% recommending this option and 41% still choosing half‐dose anticoagulants.

In the second scenario (E39–E42; Figure S6), after a further exacerbation:

- For a platelet count of 15 × 10⁹/L, 54% recommended stopping dual anti‐platelet therapy (DAPT) until platelet levels improved.
- For a platelet count of 25 × 10⁹/L, 57% favoured continuing aspirin monotherapy, and 24% chose to stop DAPT.
- For a platelet count of 35 × 10⁹/L, 35% continued DAPT.
- For a platelet count of 45 × 10⁹/L, 57% selected to continue DAPT, reflecting an increasing acceptance of dual therapy as platelet counts rose.

Overall, these treatment strategies illustrate the importance of optimising management based on platelet counts, considering the risks of thrombosis and bleeding in patients with ITP.

### 3.1 Recommendations

- The assessment of thrombotic and bleeding risks in patients with ITP should be conducted by a multidisciplinary team, including haematologists and nurses, to develop patient‐centred treatment plans.
- Bleeding risk evaluation, including the assessment of platelet count, clinical phenotype, type and dosage of antithrombotic medications, and the presence of active bleeding, is required for treatment decision‐making.
- Arterial and venous thrombotic risk factors should be reassessed regularly, especially during high‐risk events (e.g., surgery), considering thromboprophylaxis even in patients with thrombocytopenia.
- Newly diagnosed patients with ITP should be screened for APS as it may influence treatment choices.
- During a thrombotic event, effective ITP therapy should not be automatically stopped to avoid rebound thrombocytopenia and ensure the maintenance of a haemostatic platelet count to facilitate any necessary anticoagulation/antiplatelet therapy.
- It is important to involve patients in treatment decisions, taking their preferences into account.
- Ongoing education for healthcare professionals and patients is crucial, particularly regarding thrombotic and bleeding risk management in patients with ITP.

## 4 Discussion

This study reports the consensus amongst UK‐based haematologists and ITP specialists with 97% (*n* = 33/34) of statements reaching consensus agreement.

While treatment with antiplatelet and anticoagulation is considered effective in preventing thrombosis, currently there are no specific guidelines for managing thrombosis in patients with ITP [12], and oncology guidelines for cancer‐associated venous thrombosis are often adapted in practice which can be problematic as clinical factors and bleeding risk differ [7, 22, 25].

The results highlight some inconsistencies regarding managing thrombotic risk in patients with pITP, including uncertainty around platelet count thresholds and approach to anticoagulation and antiplatelet therapy. This reflects the current lack of guidelines and more clinical evidence may be warranted [22]. Suggestions have been made regarding antithrombotic management considering platelet thresholds, ITP treatment and bleeding. However, this requires further prospective validation [26]. Although patients with reduced platelet counts are at risk of bleeding, thrombotic complications can also be expected. Thrombotic risk factors include age, hospital admission, surgery, concurrent malignancy, pregnancy, exogenous oestrogen, obesity, smoking, hypertension, diabetes, hypercholesterolaemia and a history of VTE, ischaemic heart disease (IHD) or cerebrovascular accident (CVA) and appropriate risk assessment is needed [18].

Regarding the increased risk of thrombotic events associated with inherited thrombophilia, although not specifically included in the Delphi consensus statements (and therefore not tested with the wider expert group), the authors would recommend avoiding routine inherited thrombophilia screening in patients with ITP (in line with consensus recommendations). These tests have limited clinical utility and results do not affect management. Genomics England (GE) and the British Society for Haematology (BSH) published criteria and recommendations for testing for thrombophilia with a likely monogenic cause [27]. These recommend that genetic testing should only be performed if the results will directly impact patient management, and a 3 months wait after an acute thrombosis event should be observed before testing [27]. Also, genetic testing to predict a first occurrence of venous thrombosis is not recommended [27].

While specific anticoagulant selection was not included in the Delphi consensus statements, the authors would recommend using a direct oral anticoagulant (DOAC) in preference to VKA in patients with ITP and a clinical indication for anticoagulation who do not have an additional specific indication for warfarin (e.g. mechanical heart valve, triple positive APS [28]).  Low molecular weight heparin (LWMH) may be preferable for early outpatient treatment while platelet counts are unpredictable but once some stability is achieved, DOACs are typically preferred by patients due to their convenience and lack of drug and dietary interactions. DOACS are also associated with a lower risk of major and fatal haemorrhage compared to VKA (particularly intracranial haemorrhage [29]). In practice, the shorter half‐life of DOACs (and therefore shorter drug elimination time if stopped) can be helpful in the event of a fall in platelet count or a bleeding event. Similarly, the lower dosing options for DOACs can be useful in some patients with platelet counts < 50 × 10^9^/L or bleeding issues. Heparin (predominantly LMWH) may be the preferred anticoagulant for unwell, unstable patients admitted to hospital.

Randomised placebo‐controlled trials (RCTs) of TPO‐RAs were not powered to detect clinically relevant differences in thrombosis risk. Such trials did not show significantly increased thrombosis rates, and therefore any potential risk increase is likely to be small [30].

RCTs of TPO‐RAs of patients with ITP likely underestimates thrombosis risk as they exclude patients with high risk factors [18]. These factors are common in ITP patients: a 2024 prospective study by Goncalves et al. reported that 20% of ITP patients has two or more thrombosis risk factors [18]. Occurrence of thrombosis risk factors is higher in those with chronic ITP, with long‐term safety data indicated that 58% of patients had multiple risk factors [31]. This highlights the critical need, as supported by recent literature [18, 19], for continuous assessment and management of thrombosis risk factors throughout an ITP patient's history. These complexities are exacerbated by the need to balance bleeding risk with thrombosis. A 2024 practical guide to the management of ITP, in line with the results from this consensus, recommended that the evaluation of these patients requires a multidisciplinary team to individualise management alongside evaluating risks and benefits to treatment decisions [19].

Broadly, more thromboembolic events (TEEs) have been reported in patients treated with TPO‐RAs compared to those not receiving TPO‐RAs. However, meta‐analyses and network meta‐analyses (NMAs) show that the difference in risk is not statistically significant [12, 30, 32, 33, 34]. Patients with ITP have slightly higher annual thrombosis rates, and some non‐randomised studies reported a modestly higher risk with TPO‐RAs compared to immunosuppressants [35]. Long‐term TPO‐RA studies estimate a 6%–7% incidence of thrombotic events across agents, with no clear link to platelet counts [36, 37, 38]. No head‐to‐head trials have compared TPO‐RAs, and the French Pharmacovigilance Database found no significant risk differences between eltrombopag and romiplostim [39, 40]. In older individuals, a small but significant increase in venous thromboembolism was reported with TPO‐RA use [35]. There is no substantial evidence to indicate differences in thrombogenicity among TPO‐RAs [39].

Low agreement exhibited for S19 may be due to the specific wording of the statement used, as high platelet counts are associated with increased thrombotic risk. This could have been more accurately written as ‘From available clinical trial data, there is no clear correlation between platelet counts and thrombotic risk on TPO‐RA treatment.’

Many patients with ITP, including those with comorbidities such as atrial fibrillation, venous or arterial thromboembolism, myocardial infarction, or stroke may also require anticoagulation treatment [41]. Emerging evidence recommends a stepwise approach to anticoagulation treatment. For all ITP patients with a platelet count ≥ 50 × 10^9^/L, initiating anticoagulation treatment at the standard therapeutic dose is recommended. For those with a platelet count 25–50 × 10^9^/L, treatment with LWMH at half‐therapeutic dosage may be preferred (or prophylactic dose DOAC) [41].

Strategies for patients with ITP who develop acute proximal DVT and present with different platelets counts remain uncertain, there is no ‘one‐size‐fits‐all’ approach [42]. Evidence suggests stopping antithrombotic therapy if the platelet count is < 30 × 10^9^/L and restarting once the platelet count increases to 30–50 × 10^9^/L [19]. These results demonstrate that treatment choices depend on individual cases, with HCPs performing a more liberal approach to anticoagulation therapies, including aspirin.

Antiphospholipid antibodies, including anti‐cardiolipin antibodies and lupus anticoagulant, are prevalent and can be detected in approximately 46% of patients with ITP [7, 43]. Routine screening of patients with ITP for APS has not previously been recommended unless specific clinical criteria are present (thrombotic or obstetric) [7]. However, positive APL serology is associated with thrombotic risk and may influence subsequent therapy choices for ITP, and therefore, testing may be helpful. This reflects the agreement amongst respondents (B16, 85%) [7, 43, 44].

Shared decision‐making has become a topic of increased focus in the management of ITP [23] and a toolkit co‐developed by HCPs and patients is available [24]. Results indicate that clinicians agree that patients should be involved in their treatment decisions, as these patients may be on multiple medications for management of prothrombotic co‐morbidities, so factors such as administration and monitoring requirements may be of importance to some [45, 46, 47].

There is a need to educate patients about treatment goals and available therapeutic options, which could help improve patients’ outcomes. Patients should also be informed about the signs and symptoms of potential adverse events and the steps for proactive prevention and control [7]. Several programs in the UK, such as the Haematology Nurses & Healthcare Professionals Group and the ITP Support Association, provide education and support for healthcare professionals and patients.

### 4.1 Strengths and Limitations

The strength of this study is the high level of agreement demonstrated by a representative cohort of haematologists from the UK in almost all statements. Respondents clearly recognise and agree with proposed statements. However, these high agreement levels may indicate a lack of challenge or a bias towards agreeability. In addition, consensus does not equate to evidence from randomised or observational studies and therefore cannot ensure generalisability. Retrospectively, the respondent group inclusion criteria could have included specific indicators of experience and/or expertise. The panel included a well‐balanced representation from a wide range of Trusts, including specialist and non‐specialist centres. However, results are biased towards practice in England, as the majority of respondents were based there (*n* = 41/47), recommendations may therefore not be directly applicable in other devolved UK nations due to differences in service design and delivery.

## 5 Conclusions

This modified Delphi consensus study established the strength of agreement amongst haematologists towards the management of pITP which supports the proposed recommendations to improve management of thrombotic risk in pITP. The management and reassessment of thrombotic and bleeding risks require a collaborative, multidisciplinary approach which considers patient preference. Individual platelet count, clinical presentation and the risk of rebound thrombocytopenia should all be considered when adjusting therapy. It is hoped that these findings provide practical advice for the management of thrombotic risk in individuals with pITP.

## Author Contributions

Charlotte Bradbury, Will Lester and Jecko Thachil developed the initial statements, contributed to the analysis and discussion of results equally and edited and approved the final manuscript. Matthew McWilliams substantially contributed to study conception and design as well as technical accuracy review of manuscript.

## Ethics Statement

The authors have nothing to report.

## Consent

The authors have nothing to report

## Conflicts of Interest

All authors received honoraria from Sobi while undertaking this study. Sobi commissioned Triducive Partners Limited to facilitate the project and analyse the responses to the consensus statements in line with the Delphi methodology. CB has received speaker fees from Bristol Myers Squibb/Pfizer Alliance, Novartis, Lilly, Bayer, Sanofi, Sobi and Janssen, has been supported to attend conferences by Amgen, Bayer, Sanofi and Novartis, is an advisor for Novartis, Ablynx, Lilly and Bristol Myers Squibb/Pfizer Alliance. JT has received Amgen Novartis, and Sobi. MM is an employee of SOBI Ltd. WL has received speaker and advisory board for Grifols, Takeda, Roche and Novartis.

## Supporting information



Supporting File: jha270134‐sup‐0001‐SuppMat.docx

**Supporting Figure S1**: Respondent time in role.

**Supporting Figure S2**: Respondents by country.

**Supporting Figure S3**: Respondents by centre ID.

**Supporting Figure S4**: Percentages of agreement level by statement (Eight scenario statements have been analysed separately).

**Supporting Figure S5**: Analysis of scenario statements in Domain D.

**Supporting Figure S6**: Analysis of scenario statements in Domain D.

## Data Availability

Data available upon written request from Triducive Partners Ltd.
