# Supplementary material for: Appropriate Management of Thrombotic Risk in Patients With Primary Immune Thrombocytopenia in the UK: A Modified Delphi Consensus
Source: EJHaem. 2025 Sep 3;6(5):e70134. doi: 10.1002/jha2.70134 (PMC12406081; doi:10.1002/jha2.70134)
Supplement: Supplementary file 1 — Supporting File: jha270134‐sup‐0001‐SuppMat.docx [file JHA2-6-e70134-s002.docx]

**SUPPLEMENTARY INFORMATION**

**Supplemental Figure S1.** Respondent time in role.

**Supplemental Figure S2.** Respondents by country.

**Supplemental Figure S3.** Respondents by centre ID.

**Supplemental Figure S4.** Percentages of agreement level by statement (Eight scenario statements have been analysed separately).

**Supplemental Figure S5.** Analysis of scenario statements in Domain D.

**Supplemental Figure S6.** Analysis of scenario statements in Domain D.
