# Supplementary figures and images for: Appropriate Management of Thrombotic Risk in Patients With Primary Immune Thrombocytopenia in the UK: A Modified Delphi Consensus
Source: EJHaem. 2025 Sep 3;6(5):e70134. doi: 10.1002/jha2.70134 (PMC12406081; doi:10.1002/jha2.70134)

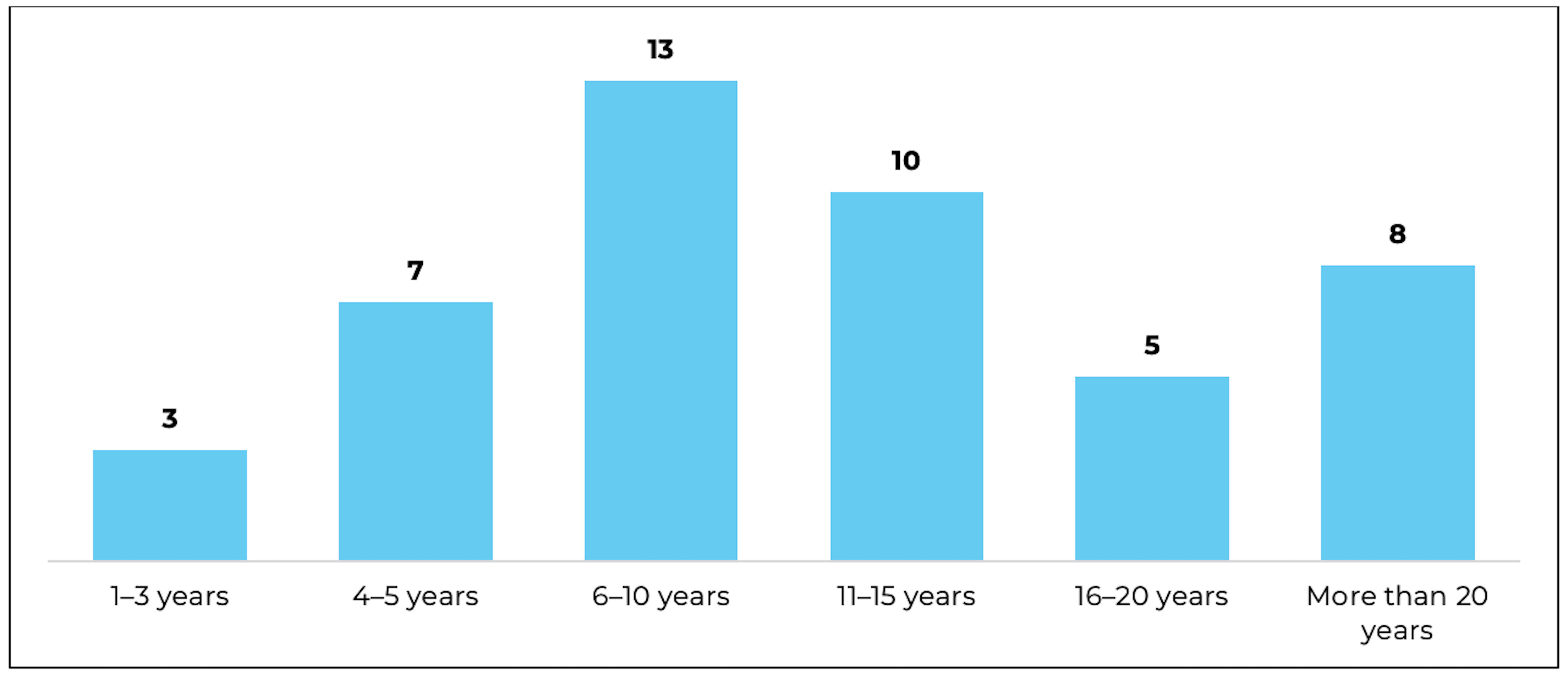

Supplement: Supplementary file 2 — Supporting Figure S1: Respondent time in role. [file JHA2-6-e70134-s006.jpeg]

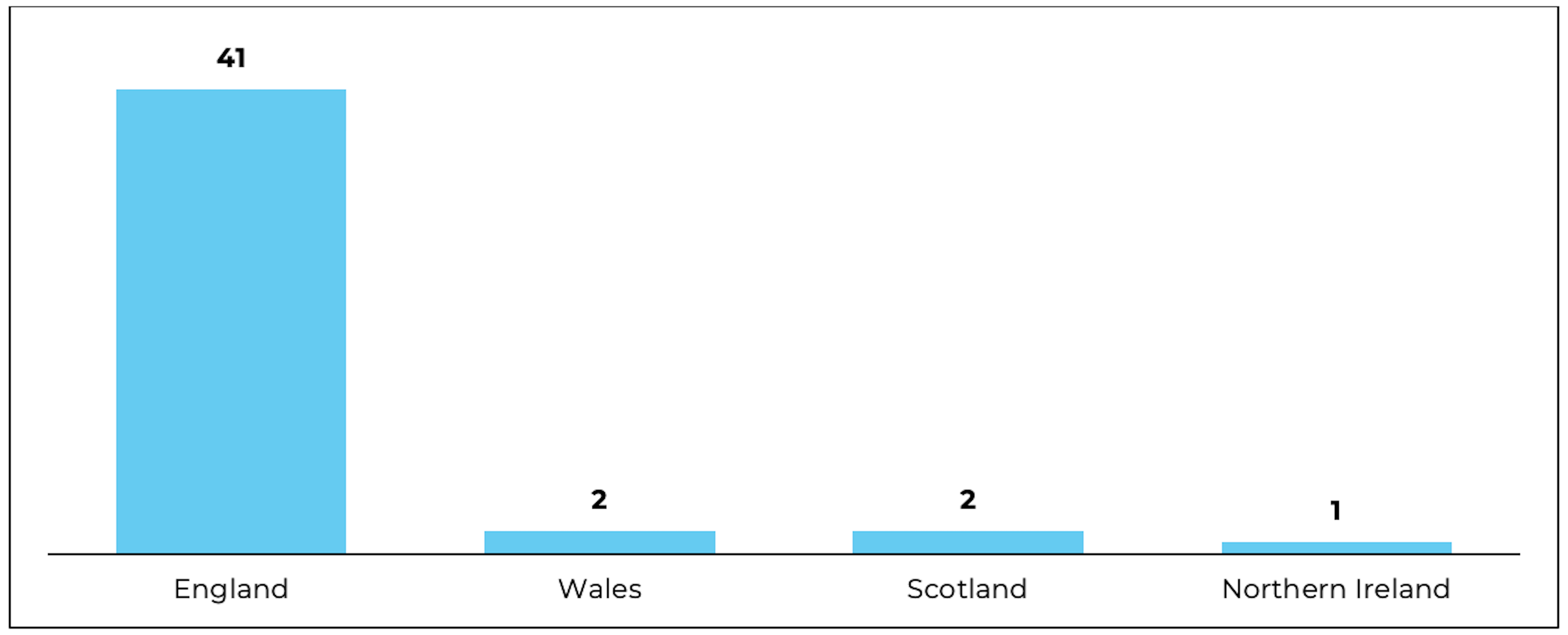

Supplement: Supplementary file 3 — Supporting Figure S2: Respondents by country. [file JHA2-6-e70134-s005.jpeg]

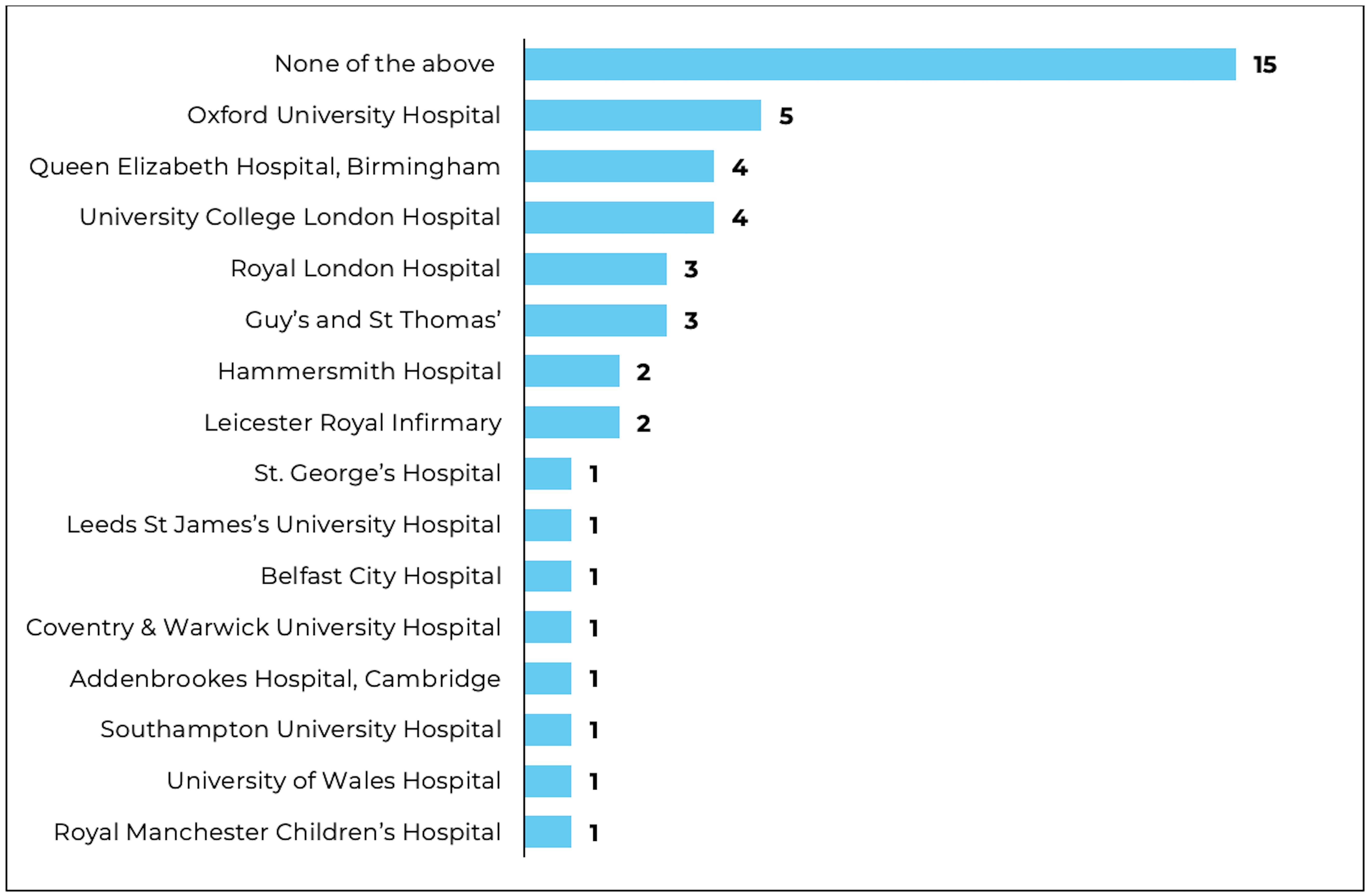

Supplement: Supplementary file 4 — Supporting Figure S3: Respondents by centre ID. [file JHA2-6-e70134-s003.jpeg]

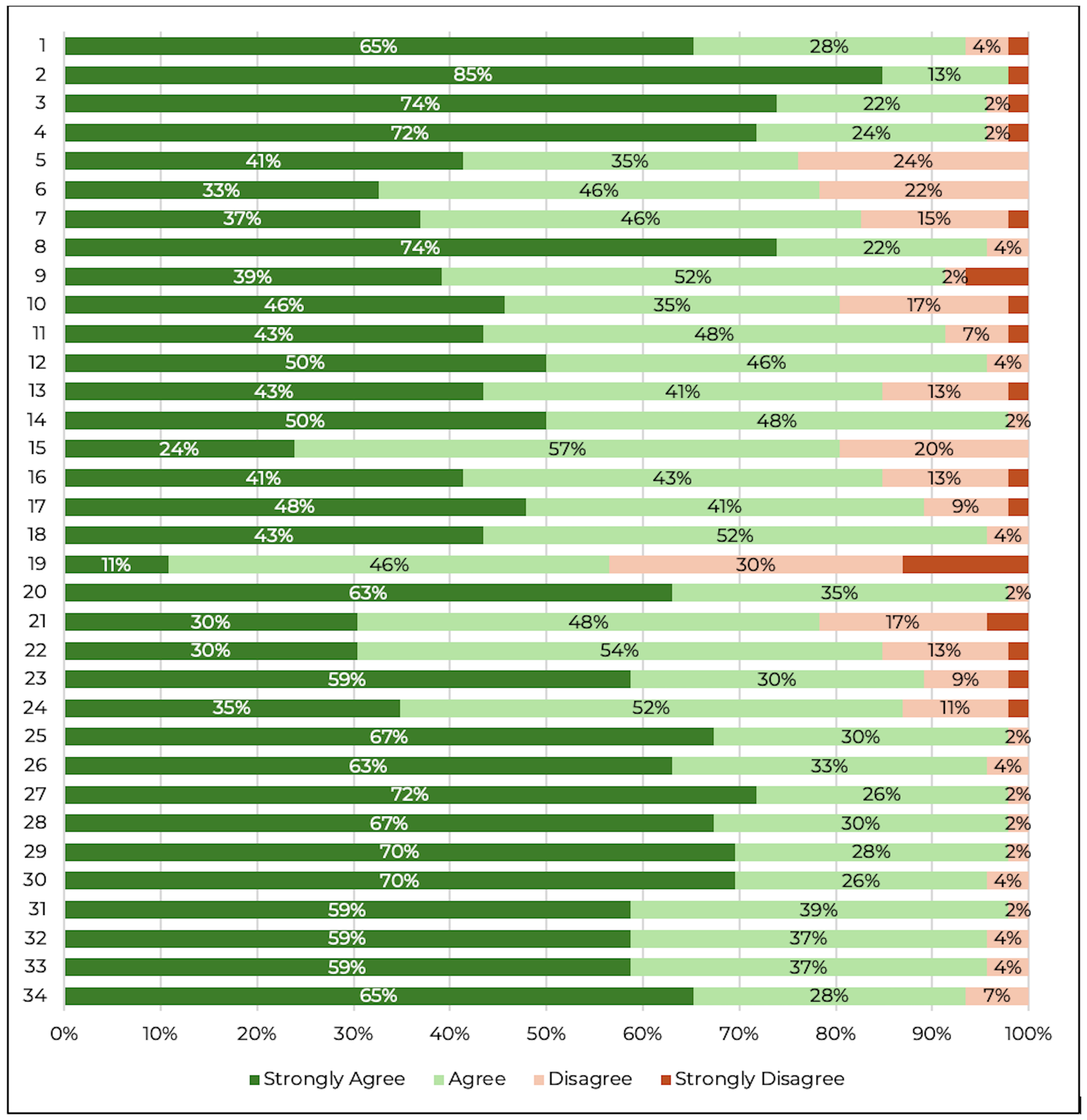

Supplement: Supplementary file 5 — Supporting Figure S4: Percentages of agreement level by statement (Eight scenario statements have been analysed separately). [file JHA2-6-e70134-s001.jpeg]

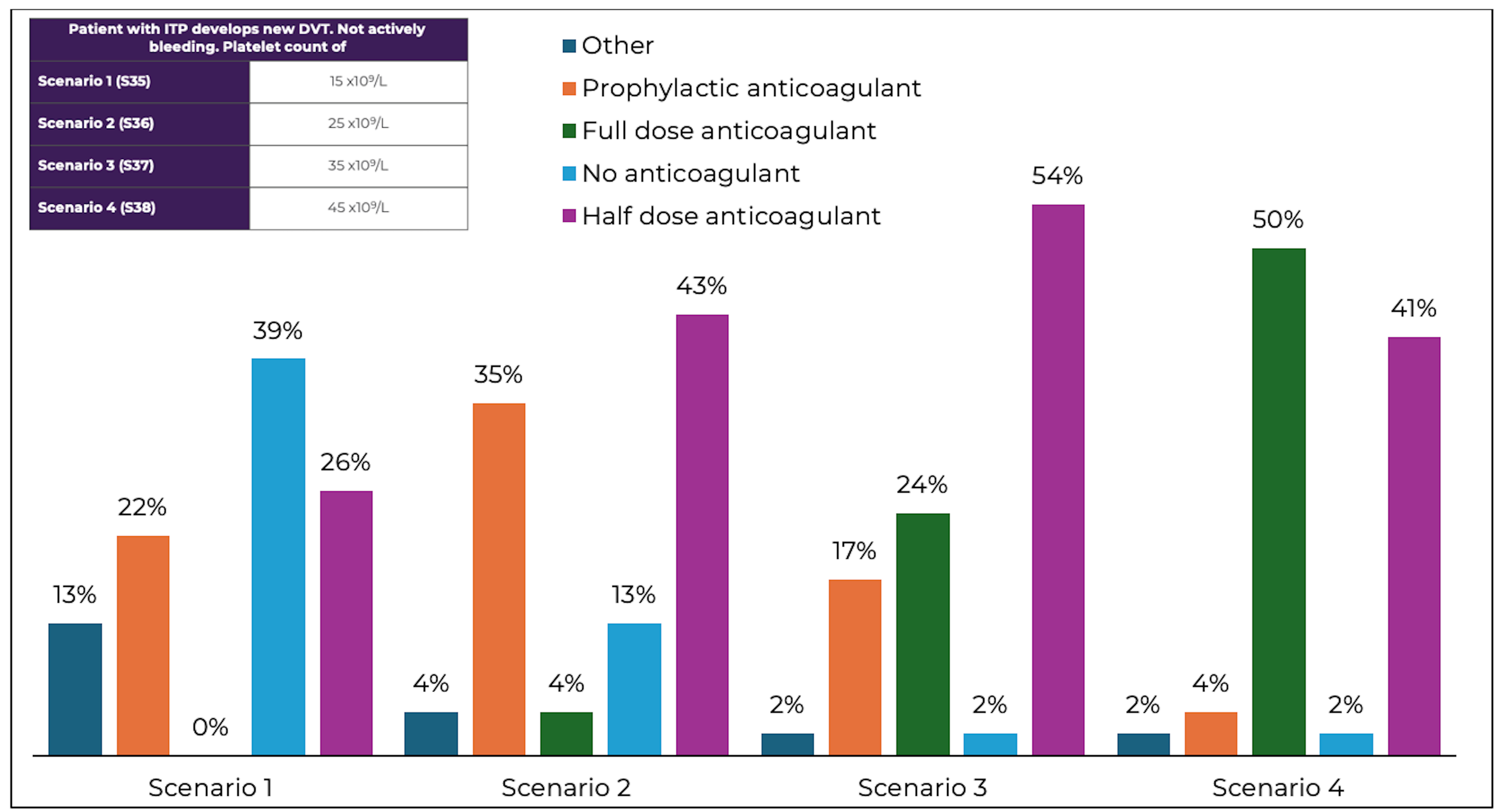

Supplement: Supplementary file 6 — Supporting Figure S5: Analysis of scenario statements in Domain D. [file JHA2-6-e70134-s007.jpeg]

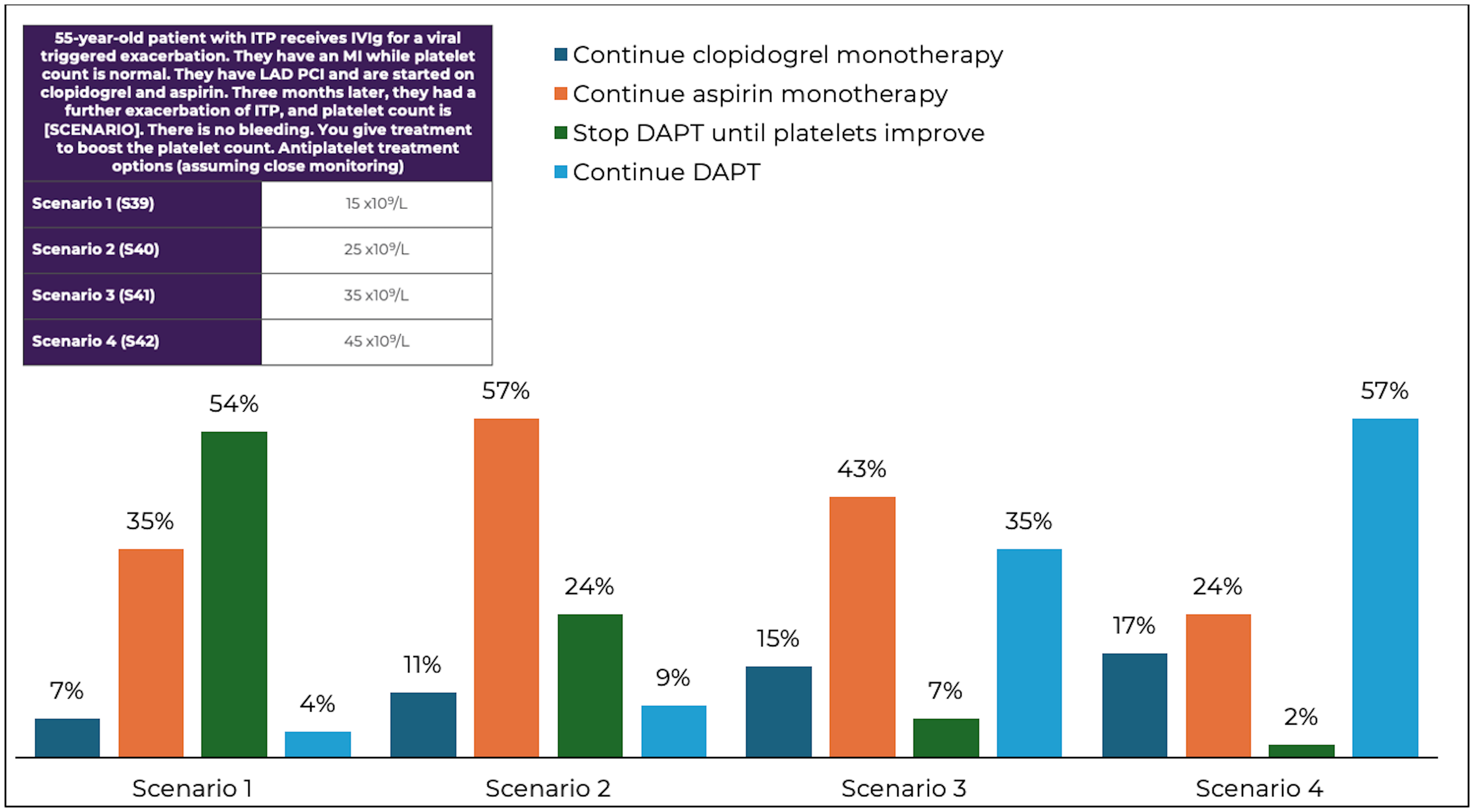

Supplement: Supplementary file 7 — Supporting Figure S6: Analysis of scenario statements in Domain D. [file JHA2-6-e70134-s004.jpeg]
